# Development of prostate specific membrane antigen targeted ultrasound microbubbles using bioorthogonal chemistry

**DOI:** 10.1371/journal.pone.0176958

**Published:** 2017-05-04

**Authors:** Aimen Zlitni, Melissa Yin, Nancy Janzen, Samit Chatterjee, Ala Lisok, Kathleen L. Gabrielson, Sridhar Nimmagadda, Martin G. Pomper, F. Stuart Foster, John F. Valliant

**Affiliations:** 1 Department of Chemistry and Chemical Biology, McMaster University, Hamilton, Ontario, Canada; 2 Department of Medical Biophysics, University of Toronto, Toronto, Ontario, Canada; 3 Russell H. Morgan Department of Radiology and Radiological Science, Johns Hopkins Medical Institutions, Baltimore, Maryland, United States of America; 4 Centre for Probe Development and Commercialization, Hamilton, Ontario, Canada; Monash University, AUSTRALIA

## Abstract

Prostate specific membrane antigen (PSMA) targeted microbubbles (MBs) were developed using bioorthogonal chemistry. Streptavidin-labeled MBs were treated with a biotinylated tetrazine (MB_Tz_) and targeted to PSMA expressing cells using *trans*-cyclooctene (TCO)-functionalized anti-PSMA antibodies (TCO-anti-PSMA). The extent of MB binding to PSMA positive cells for two different targeting strategies was determined using an *in vitro* flow chamber. The initial approach involved pretargeting, where TCO-anti-PSMA was first incubated with PSMA expressing cells and followed by MB_Tz_, which subsequently showed a 2.8 fold increase in the number of bound MBs compared to experiments performed in the absence of TCO-anti-PSMA. Using direct targeting, where TCO-anti-PSMA was linked to MB_Tz_ prior to initiation of the assay, a 5-fold increase in binding compared to controls was observed. The direct targeting approach was subsequently evaluated *in vivo* using a human xenograft tumor model and two different PSMA-targeting antibodies. The US signal enhancements observed were 1.6- and 5.9-fold greater than that for non-targeted MBs. The lead construct was also evaluated in a head-to-head study using mice bearing both PSMA positive or negative tumors in separate limbs. The human PSMA expressing tumors exhibited a 2-fold higher US signal compared to those tumors deficient in human PSMA. The results demonstrate both the feasibility of preparing PSMA-targeted MBs and the benefits of using bioorthogonal chemistry to create targeted US probes.

## Introduction

Prostate cancer (PCa) is the second leading cause of cancer-related deaths in men [1]. It is estimated that in 2015 more than 220,000 people in the USA and 24,000 in Canada were diagnosed with PCa, which would account for 26% of all new cancer cases in men [1,2]. When detected early, the 5-year survival rate is around 99%. However, survival drops dramatically once the cancer has spread beyond the prostate [3]. The gold standard for PCa diagnosis is trans-rectal ultrasound (TRUS)-guided biopsies from patients with elevated serum levels of prostate specific antigen. Unfortunately, TRUS biopsies have a high rate of false-negative results often leading to repeat biopsy procedures [4–7]. While ultrasound (US) imaging of PCa is used routinely, its role in visualizing focal lesions is limited by the lack of targeted imaging [8]. This is driving the need for PCa-targeted US contrast agents to enhance the sensitivity and accuracy of US in detecting malignant masses both during diagnosis and following the initiation of therapy [9]. Such a technique would help improve diagnostic sensitivity and specificity, help support active surveillance strategies and reduce the need for repeat biopsies [9]. Although recent work has indicated that magnetic resonance imaging (MRI) guided biopsy may be superior to TRUS in detection of clinically significant PCa [10], the availability, portability and relative low cost of US make it an attractive tool for active surveillance of PCa particularly when combined with targeted contrast agents.

One approach to improving PCa detection is to employ imaging agents that target prostate specific membrane antigen (PSMA). PSMA is a transmembrane glycoprotein that is expressed at low levels in normal prostate, liver, kidney and brain tissue, but is expressed at much higher levels in PCa tumors [11–17]. High PSMA expression correlates with pathological stage and tumor grade, and was demonstrated as an independent predictor of biochemical recurrence [18,19]. Agents for visualizing PSMA with nuclear imaging methods such as positron emission tomography (PET) include both radiolabeled small molecules and antibodies, which have been used clinically in patients with both primary and metastatic disease [20–30]. Results from these studies support the use of PSMA as a PCa biomarker.

A microbubble based (MB) contrast agent that targets PSMA would provide the opportunity to use US imaging to detect and characterize primary and recurrent PCa [31]. PSMA is highly expressed on the endothelial cells in the microvasculature of prostate tumors making it a suitable target for molecular US imaging using targeted MBs, which are generally restricted to targets within the vasculature due to their size [32]. A PSMA-targeted US method could be used for detecting PCa lesions and for biopsy guidance, offering a way to improve existing non-contrast enhanced US techniques, while also providing an alternative to more costly and time consuming MRI-based biopsy guidance methods [33].

Sanna and coworkers prepared polymer-based MBs covalently attached to a small-molecule inhibitor of PSMA [34]. The MBs showed specific binding to PSMA-expressing (PSMA^+^) cells *in vitro*, however the evaluation of this agent under dynamic flow conditions or in preclinical animal models has not yet been reported. Wang and coworkers prepared nano-scale US contrast bubbles (NBs) coated with streptavidin and loaded with a biotinylated derivative of an anti-PSMA antibody [35]. *In vitro* and *in vivo* studies showed a statistically significant but modest difference in the binding of the PSMA-targeted NBs, where the ratio of the US signal obtained from targeted compared to non-targeted NBs was less than 1.20. These researchers also targeted the same NBs using a biotinylated derivative of an anti-PSMA nanobody, which showed similar binding compared to the anti-PSMA antibody [36]. Although this prior work indicates targeting to PSMA may be feasible, it is clear that the type of targeting agent and the nature of its linkage to the US contrast agent is critical and have not been fully optimized. Furthermore, the methods used to produce the PSMA-targeted MBs should be readily accessible to the scientific community and the associated chemistry adaptable for producing MBs that can be used in clinical studies.

**Fig 1:**
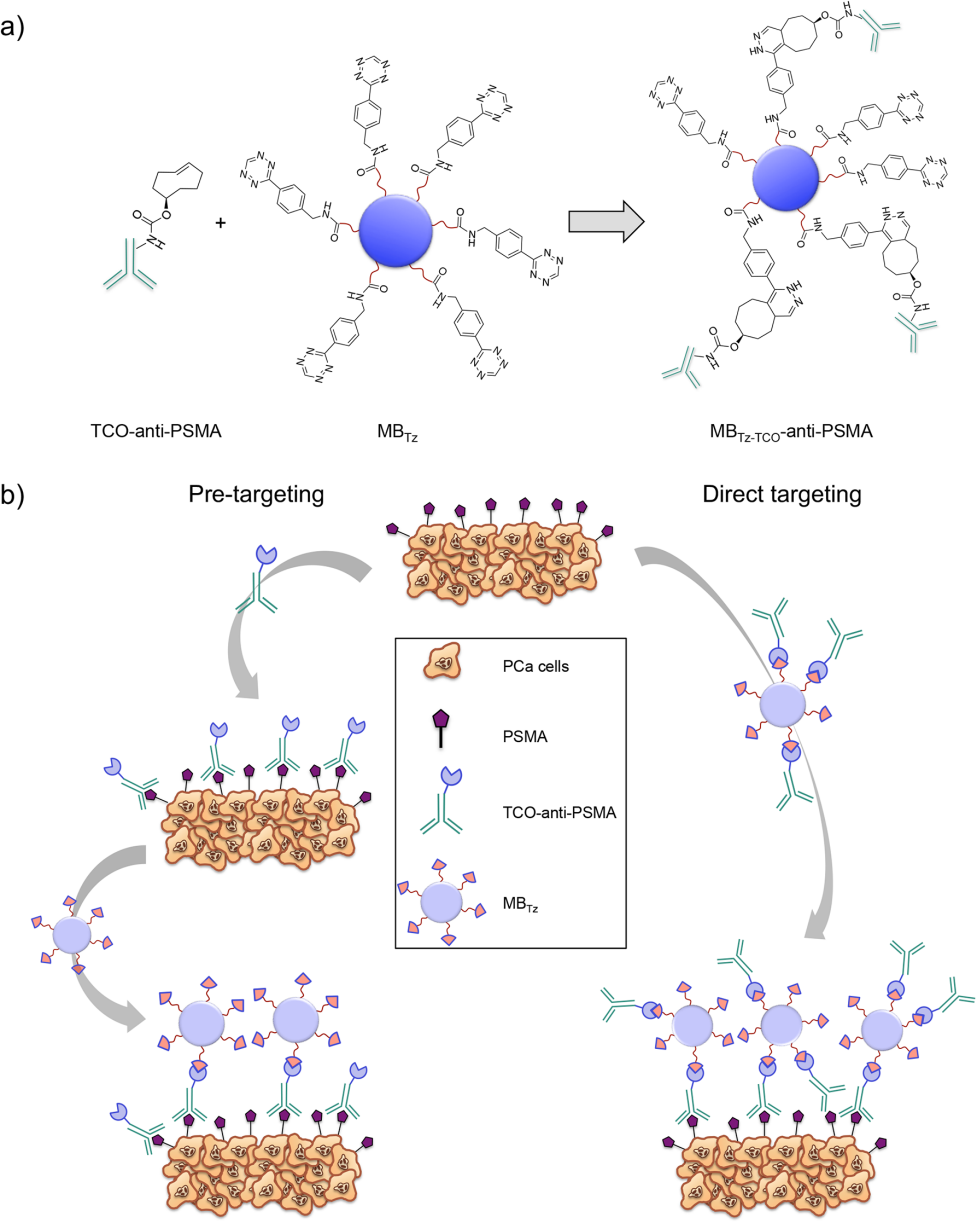
Cycloaddition reaction and targeting strategies for MB_Tz_. a) Schematic illustrating the cycloaddition reaction between tetrazine functionalized microbubbles (MB_Tz_) and *trans*-cyclooctene (TCO)-conjugated PSMA antibody (TCO-anti-PSMA) producing targeted MBs (MB_Tz-TCO_-anti-PSMA). b) Schematic representation of the two strategies used to target MB_Tz_ to PSMA-expressing PCa cells. Right: MB_Tz_ is treated with TCO-anti-PSMA (MB_Tz-TCO_-anti-PSMA) before injection (direct targeting). Left: TCO-anti-PSMA is injected first and allowed to bind to PSMA-expressing cells and clear non-targeted tissue before injecting MB_Tz_ (pretargeting).

Here we utilize the high yielding, selective and rapid bioorthogonal chemical reaction between tetrazine (Tz) and *trans*-cyclooctene (TCO) to create PSMA binding MBs (Fig 1A) [37]. This methodology has been used successfully to target MBs to the angiogenesis marker vascular endothelial growth factor receptor 2 (VEGFR2) [37] where the use of bioorthogonal chemistry affords the opportunity to employ two different approaches for targeting MBs to the biomarker of interest (Fig 1B). The first is a direct targeting strategy, which involves ligating a target-specific, TCO-labeled antibody (TCO-anti-PSMA) with Tz-functionalized MBs (MB_Tz_) creating antibody-targeted MBs (Fig 1A), prior to administration and US imaging. The second is a pretargeting strategy, in which the TCO-labeled antibody is administered first, to allow binding to sites of target antigen overexpression and clearance from non-target organs. This is followed by injection of MB_Tz_ that will selectively react with target-bound TCO-anti-PSMA *in vivo*. While the former has the advantage of simplicity, the latter eliminates the influence of the large size of the MB on the ability of the antibody to bind the target. We report here the development and evaluation of both approaches and demonstrate US imaging of PSMA-expressing tumors *in vivo*.

## Materials and methods

### General materials and instruments

Microbubbles (MBs) were obtained (8.4 × 10^8^ MBs/vial) from a MicroMarker^TM^, Target-Ready Contrast Agent Kit (VisualSonics Inc., Toronto, ON). Streptavidin coated magnetic beads (New England BioLabs, Whitby, ON) and MACSiMAG^TM^ separator magnet (Miltenyi Biotec, Auburn, CA) were used during the purification of MBs. Conjugated antibodies were analyzed by mass spectrometry on a MALDI Bruker Ultraflextreme Spectrometer. MB size and concentration were determined using Z2 Coulter counter (Beckman Coulter, Fullerton CA). The syringe pump used in the flow chamber assay was a PhD 2000 (Harvard Apparatus, Holliston, MA). Western blot images were generated using a STORM 840 imaging system (GMI Ltd., Ramsey, MN)

### Preparation of TCO-modified antibodies

J591, an anti-PSMA monoclonal antibody (mAb) that binds the extracellular domain of human PSMA [38], was provided by Dr. Neil Bander (Department of Urology, Cornell University), where TCO-modified J591 was prepared following a modified literature method [37]. Briefly, the pH of a solution of J591 antibody (500 µL, 250 μg, 1.67 nmol) in PBS was adjusted to 9 by adding 3 µL of 1 M Na_2_CO_3_ (aq). (*E*)-Cyclooct-4-enyl-2,5-dioxopyrrolidin-1-yl carbonate (TCO-NHS) was then added (17.8 μg, 66.8 nmol, 40 eq) in 9 μL of DMSO. The solution was left on a shaker overnight at 4°C. The desired product (TCO-J591) was isolated from excess TCO using an Amicon Ultra-0.5 Centrifugal filter (30 kDa) and washed with PBS three times. Matrix-Assisted Laser Desorption Ionization Time-of-Flight Mass Spectrometry (MALDI-TOF MS) analysis of the antibody, before and after conjugation to TCO, showed an average of 1.2 TCO groups per antibody using a MALDI Bruker Ultraflextreme Spectrometer (S1 File). TCO was added to a second PSMA binding antibody (ARP44691_p050; Aviva Systems Biology, San Diego, CA) following the same method where the product is referred to herein as TCO-ARP.

### Synthesis of biotin-Tz

*N*-(4-(1,2,4,5-Tetrazin-3-yl)benzyl)-6-(5-((4*S*)-2-oxohexahydro-1*H*-thieno[3,4-*d*]imidazol-4-yl)pentanamido)hexanamide (biotin-Tz) was synthesized as previously described [37]. The structure of biotin-Tz is shown in S2 File.

### Preparation of tetrazine-functionalized microbubbles (MB_Tz_)

Streptavidin-coated MBs (MicroMarker^TM^; VisualSonics Inc.) were reconstituted in 500 μL sterile saline (0.9% NaCl), according to the manufacturer’s instructions. To prepare MB_Tz_, biotin-Tz (70 µg, 1.35 × 10^−4^ mmol) in 50 µL of saline:methanol (1:1 v/v) was added dropwise to the reconstituted MBs. After 45 min, 200 μL from the bottom of the vial was removed carefully with minimal agitation and was discarded. Then, a 200 μL suspension of streptavidin-coated magnetic beads (New England BioLabs) were added and the solution set aside for 20 min. Thereafter, 200 μL of the solution was removed carefully and discarded, and the remaining mixture was placed beside a MACSiMAG^TM^ magnet (Miltenyi Biotec) to remove any residual magnetic bead-bound biotin-Tz. Saline (200 μL) was then added to MBs and the solution transferred to another vial. MBs size and concentration were determined using Z2 Coulter counter (Beckman Coulter).

### Preparation of anti-PSMA antibody coated MBs

Anti-PSMA coated MBs were prepared by combining MB_Tz_ with either TCO-J591 or TCO-ARP. Briefly, a MB_Tz_ solution (50 µL, 3 × 10^7^ MBs for *in vitro* studies; 120 µL, 7 × 10^7^ MBs for *in vivo* studies) was mixed with the TCO-anti-PSMA antibody (20 µL/10 µg for *in vitro* studies; 50 µL/ 25 µg for *in vivo* studies). The reaction was allowed to proceed for 20 min at room temperature. The resulting constructs MB_Tz-TCO_-J591 and MB_Tz-TCO_-ARP were used immediately for *in vitro* binding and *in vivo* imaging studies.

### Cell lines and culture methods

PC-3 cells transfected with human PSMA were generously provided by Molecular Insight Pharmaceuticals, Inc. (Cambridge, MA). PC-3 cells were cultured in F12-K media supplemented with 10% FBS, 1% penicillin-streptomycin and 0.1% geneticin. LNCaP cells, derived from lymph node metastases of human prostate carcinoma, were purchased from ATCC (CRL-1740), and cultured in RPMI-1640 Medium supplemented with 10% FBS and 1% penicillin-streptomycin. The cell lines were maintained at 37°C under 5% CO_2_.

### Flow chamber assay

The flow assay was performed as previously described [37]. Briefly, 8 × 10^5^ of PC-3 cells were plated separately in 30 mm Corning tissue culture dishes, 2 days prior to running the assay. The setup of the parallel-plate flow chamber (Glycotech, Rockville, MD) is shown in S3 File. In the pretargeting strategy and associated controls, cells were incubated first with TCO-J591 (30 μg/mL) for 30 min. For direct targeting, TCO-J591 was incubated with MB_Tz_ for 20 min. creating the targeted MBs (MB_Tz-TCO_-J591) that were subsequently used in the flow assay. Using a syringe pump, cells were first rinsed with 1 mL of PBS, and then 1 mL of MB solution at a shear rate of 100 sec^-1^ (flow rate = 0.164 mL/min). Thereafter, the plate was rinsed with 2 mL of PBS at a shear rate of 1000 sec^-1^. Binding of MBs was visualized using a Celestron PentaView LCD Digital Brightfield S4 Microscope with 20× objective. Images were recorded and the extent of binding was assessed by comparing the area covered by MBs to the total area covered by cells in each image using image analysis (FIJI) software [37,39].

### Mouse xenograft tumor model and procedures

All research that involved in vivo study in mice was done with the approval of Institutional Animal Care and Use Committees at McMaster University (Animal Research Ethics Board), Sunnybrook Research Institute (SRI Animal Care Committee), and Johns Hopkins Medical (Animal Care and Use Committee). Mice were maintained with 12 h light/dark cycles and given food and water *ad libitum*. The human prostate carcinoma LNCaP cells were used to provide xenograft tumors. NCr nude male mice (4 to 5 week old) were purchased (Taconic Labs, Germantown, NY) and were injected with 2.0 × 10^6^ LNCaP cells in 100 µL Matrigel/DPBS (1:1; VWR-Canlab, Mississauga, ON and Invitrogen, Burlington, ON) subcutaneously in the right flank. To isolate tumor tissue, mice were sacrificed by cervical dislocation immediately following imaging, and the tumors excised, rinsed with PBS and frozen in liquid N_2_. Frozen tumors were stored at -80°C. Animal studies involving PC-3 cells utilized human prostate cancer PC-3 cells transfected to overexpress PSMA (PSMA^+^) or transfected with the plasmid alone (PSMA^–^) were implanted subcutaneously in severe-combined immunodeficient (SCID) mice (Johns Hopkins Immune Compromised Animal Core) at the forward right and left flank respectively, as previously reported [40].

### Tumor lysate preparation

Each frozen tumor was thawed and put into lysis buffer containing 1% IGEPAL CA-630 (I3021; Sigma-Aldrich, Oakville, ON), 20mM Tris pH 8.0 (154563; Sigma-Aldrich), 137mM NaCl (S6191; Sigma-Aldrich), 10% glycerol (5350–1; Caledon Laboratories), 2mM EDTA (E5134; Sigma-Aldrich) and Protease Inhibitor cocktail (PIC003; Bioshop Canada, Burlington, ON). Each tumor was subsequently homogenized using VWR PowerMax AHS 200 homogenizer (5 × 75 mL troemner) and lysate collected after centrifugation (2000 × g, 5 min) and washing three times with PBS. Protein concentration was determined using a Pierce® BCA Protein Assay Kit (ThermoFisher Scientific, Ottawa, ON).

### Western blot analysis of cell lysates

Using immunoblotting, PSMA protein expression by PSMA^+^ LNCaP and transfected PSMA^+^ PC-3 cells was assessed and compared to PSMA^-^ PC-3 cells. 10 µg of protein from each cell lysate were loaded on 10% Mini-PROTEAN TGX precast gels and fractionated by SDS-PAGE. After electro-transferring the protein extracts to polyvinylidene difluoride (PVDF) membrane, the membrane was incubated with PSMA antibody (E-18), a goat polyclonal antibody (sc-10269; Santa Cruz Biotechnology, Dallas, TX), specific for mouse and human PSMA, in a 1:250 dilution overnight at 4°C. After washing, the membrane was incubated with AP-Bovine anti-goat IgG antibody (sc-2351; Santa Cruz Biotechnology) in a 1:2000 dilution for 1 h at room temperature. The membrane was then washed and incubated with a chemiluminescent reagent (ECF substrate, GE RPN5785) for 5 min and imaged using a STORM 840 system (GMI Ltd., Charlotte, NC).

### Western blot analysis of tumor lysates

PSMA expression by LNCaP tumors used in imaging studies was assessed by immunoblotting. Tumor lysates were loaded on 10% Mini-PROTEAN TGX precast gels and fractionated by SDS-PAGE. Protein extracts were electro-transferred to PVDF membrane. The PVDF membrane was incubated with PSMA (E-18) goat anti-PSMA antibody (sc-10269; Santa Cruz Biotechnology) in a 1:250 dilution, and anti-β-Actin (13E5) rabbit mAb (4970; Cell Signaling Technology, Beverly, MA) in a 1:2000 dilution overnight at 4°C. The membrane was then washed and incubated with AP-Bovine anti-goat IgG (sc-2351; Santa Cruz Biotechnology) plus AP-Goat anti-rabbit IgG (H+L) (111055045, Jackson ImmunoResearch) in a 1:2000 dilution for 1 h at room temperature. Finally, the membrane was washed and incubated with a chemiluminescent reagent (ECF substrate, GE RPN5785) for 5 min and imaged using a STORM 840 imaging system (GMI Ltd.). Bands for PSMA and ß-actin were quantified using ImageQuant TL software (GE Healthcare Life Sciences).

### Ultrasound imaging and analysis

Using a Vevo®2100 imaging system (VisualSonics) and a 20 MHz high-frequency solid-state transducer (MS-250; VisualSonics), LNCaP tumor-bearing mice were imaged using non-linear contrast mode. Animals were kept under anesthesia using isoflurane (4% initiation, 2% maintenance) during the imaging session. MBs were administered in a 70 µL bolus (approximately 5–6 × 10^7^ MBs) via the tail vein, using a syringe pump at a 600 µL/min injection rate. Each mouse was first injected and imaged with MB_Tz_ alone as a control, followed by a *circa* 15 min delay for the MBs to clear. This was followed by a second injection of MB_Tz_ that had been coupled to the TCO-anti-PSMA antibody. After each injection, MBs were allowed to circulate for 4 min before initiating a disruption replenishment sequence [41]. Typically 200 frames of images were acquired before initiating a 1 sec high power disruption sequence, followed by another 300 frames of image acquisition. 3D disruption sequences were applied between each injection to establish complete clearance of previously injected MBs.

US imaging studies on PSMA^+^ and PSMA^−^PC-3 tumors were conducted using Vevo®3100 imaging system (VisualSonics) and a 20 MHz high-frequency solid-state transducer (MS-250; VisualSonics) using non-linear contrast mode. MBs targeted to both human and mouse PSMA (MB_Tz-TCO_-ARP) were administered as stated above and allowed to circulate for 4 min before imaging. In random order, either PSMA^+^ then PSMA^−^tumor models were imaged using a disruption replenishment sequence.

For all studies, the differential targeted enhancement (dTE) signal was measured using a contrast quantification software analysis tool (VevoCQ™, VisualSonics). Regions of interest were determined based on the distribution of the MBs in the tumor (only vascular regions were included) and was kept constant for each animal when evaluating the different MBs. In order to distinguish and quantify the US signal coming from tumor-bound MBs, the average intensity over 200 frames acquired 5 sec after disruption (which represents the US signal coming from circulating MBs) was subtracted from the average intensity over 200 frames acquired before disruption (which represents the US signal coming from tumor-bound and circulating MBs). The signal attributed to tumor-bound MBs is then presented as parametric maps overlaid onto the non-linear contrast US images.

### Statistical analysis

Statistical analysis of the data from the flow chamber binding assay and the US imaging was performed by one-way ANOVA. The results for flow chamber binding assay were confirmed post-hoc using the Bonferroni test.

## Results and discussion

### Preparation of PSMA-targeted MBs

The tetrazine functionalized microbubbles (MB_Tz_) were prepared by adding *N*-(4-(1,2,4,5-tetrazin-3-yl)benzyl)-6-(5-((4*S*)-2-oxohexahydro-1*H*-thieno[3,4-*d*]imidazol-4-yl)-pentanamido)- hexanamide (biotin-Tz, S2 File) to commercially available streptavidin coated MBs [37]. TCO-labeled anti-PSMA antibodies were prepared by combining the protein with (*E*)-cyclooct-4-enyl-2,5-dioxopyrrolidin-1-yl carbonate (TCO-NHS) at pH 9–9.5 and 4°C overnight. The number of TCO moieties per antibody was 1.2 as determined by MALDI-TOF MS (S1 File). Initial screening studies were performed using J591, which binds to the extracellular domain of human PSMA [38] and has been shown to be a highly effective tool for targeting radioisotopes and nanomaterials to PSMA expressing tumors [14,38,42–46]. J591 was chosen from among several anti-PSMA mAbs that have been investigated for targeted imaging and targeted radiotherapy [47–52]. J591 has the advantages of being a humanized mAb used in clinical studies [28] and it binds to the extracellular domain of PSMA present on the surface of viable tumor cells [38], rather than targeting the intracellular domain that is accessible only within necrotic regions [53].

### *In vitro* screening of direct and pretargeting of MBs to PSMA

The ability to target MBs to PSMA *in vitro* was assessed in a flow chamber adhesion assay (S3 File). The flow chamber system is designed to provide a more realistic test environment for evaluating the ability of targeted MBs to bind receptors in the dynamic environment found in tumor microvasculature compared to that of a traditional cell culture assays [54]. Although the PSMA^+^ LNCaP cells were chosen for *in vivo* targeting studies (*vide infra*), they have low adhesive properties in culture, and therefore were not suitable for use in the flow chamber system. Instead, more adherent PC-3 cells that were PSMA^+^ (human PSMA gene-transfected) or PSMA^-^ (non-transfected) were used. Western blot analysis of the two PC-3 cell lines showed PSMA protein expression in the PSMA^+^ PC-3 cells at levels comparable to LNCaP cells, and no expression by PSMA^-^ PC-3 cells (S4 File).

Both the pretargeting and direct targeting approaches were evaluated using the *in vitro* flow chamber assay. For pretargeting, TCO-J591 was incubated with the PC-3 cells that were then rinsed before introducing MB_Tz_. For direct targeting, TCO-J591 was first incubated with MB_Tz_ creating the targeted MBs (MB_Tz-TCO_-J591). Both the MB_Tz_ (pretargeting) and the MB_Tz-TCO_-J591 conjugates (direct targeting) were added at a standard flow rate. After increasing the flow rates to remove any non-covalently bound MBs, bright-field microscopy images were collected and images were analyzed using FIJI software following a literature procedure [37].

**Fig 2:**
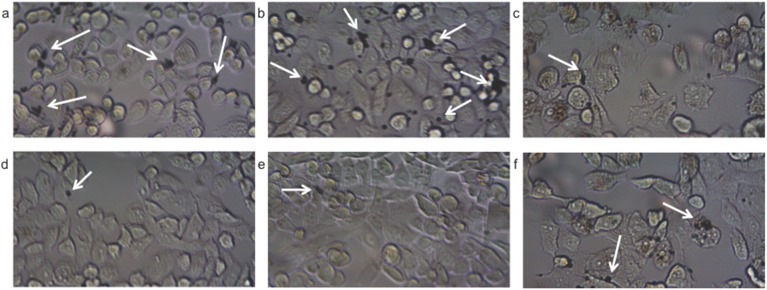
Bright-field microscopy of MB_Tz_ targeted to cell lines *in vitro*. Images are 20× and show binding of: a) MB_Tz_ to PSMA^+^ PC-3 cells pretreated with TCO-J591; b) MB_Tz_ complexed with TCO-J591 (MB_Tz-TCO_-J591) to PSMA^+^ PC-3 cells; c) MB_Tz_ to PSMA^+^ PC-3 cells with no antibody; d) MB_Tz_ to PSMA^-^ PC-3 cells pretreated with TCO-J591; e) MB_Tz-TCO_-J591 to PSMA^-^ PC-3 cells and f) Control MBs (MB_C_), which have no tetrazine present, to PSMA^+^ PC-3 cells pretreated with TCO-J591. The MBs appear as black spheres (select examples are highlighted with white arrows).

**Fig 3:**
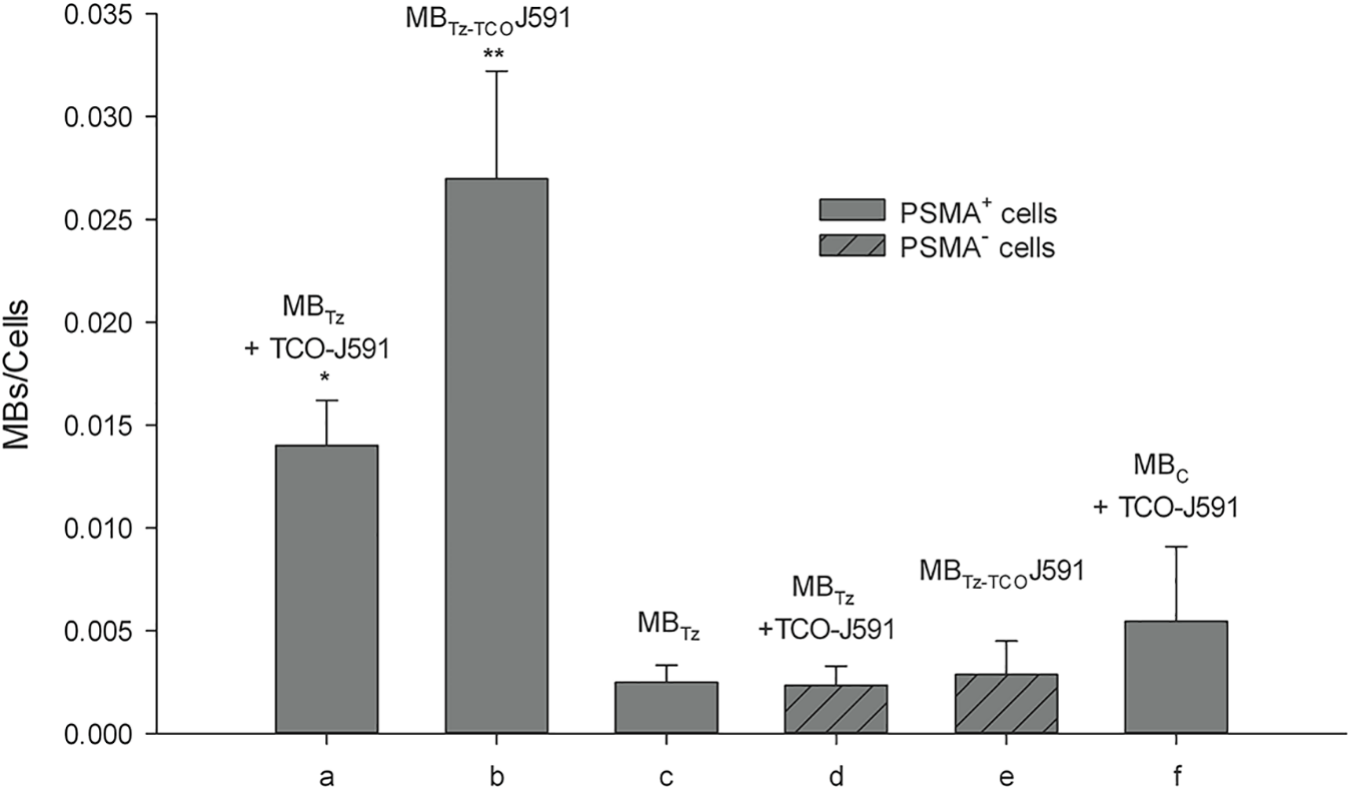
Semi-quantitative analysis of MB_Tz_ binding to cell lines *in vitro*. The number of MBs bound per cell (n = 3 replicates) were determined from bright-field microscopy images. Targeting approaches and controls are indicated (a-f): (a) pretargeting; (b) direct targeting; (c) MB_Tz_ alone (control); (d) pretargeting with PSMA^-^ PC-3 cells (control); (e) direct targeting with PSMA^-^ PC-3 cells (control); (f) pretargeting with unmodified MBs (control). Statistically significant difference for direct targeting (b) versus (c) or (e), *P* < 0.001; for pretargeting (a) versus (c), (d) or (f), *P* < 0.001. The binding of the non-conjugated, MB_C_ control (f) was not significantly different (*P* > 0.05) from other controls (c, d, and e). Statistical analysis was performed using one-way ANOVA.

Microscopy images (Fig 2) showed higher binding for both pretargeting and direct targeting compared to what was observed for non-functionalized MBs. Image analysis indicated significantly lower binding of MB_Tz_ to PSMA^+^ PC-3 cells (Fig 3) using the pretargeting approach compared to the direct targeting approach. However, binding of MB_Tz_ by pretargeting was still 2.8 times higher than controls in which non-conjugated MBs (no Tz) or PSMA^-^ PC-3 cells were used (Fig 3). For the direct targeting approach, the results showed greater than 5-fold higher binding of MB_Tz-TCO_-J591 to PSMA^+^ PC-3 cells compared to controls. The increased binding with direct targeting versus pretargeting is likely due to the internalization of the TCO-J591, which has been previously reported for J591 [55]. Based on the data, and to avoid the influence of internalization, only the direct targeting strategy was advanced for evaluation *in vivo*.

### *In vivo* targeting of MBs to PSMA in LNCaP xenografts

LNCaP cells were used to create a PSMA expressing tumor xenograft model [56] in NCr nu/nu mice [57]. Following each US imaging study, PSMA expression in the isolated tumor was verified by western blot of tumor lysates (S4 File). The amounts of PSMA expressed by all tumors used for US imaging showed little variability when normalized to β-actin expression (S4 File). As a control in all studies, non-targeted MB_Tz_ were allowed to accumulate in the tumors for 4 min before a disruption replenishment sequence was conducted [37]. After 15 min, a 3D disruption sequence was applied to ensure there were no residual MBs prior to injecting the PSMA-targeting contrast agent.

**Fig 4:**
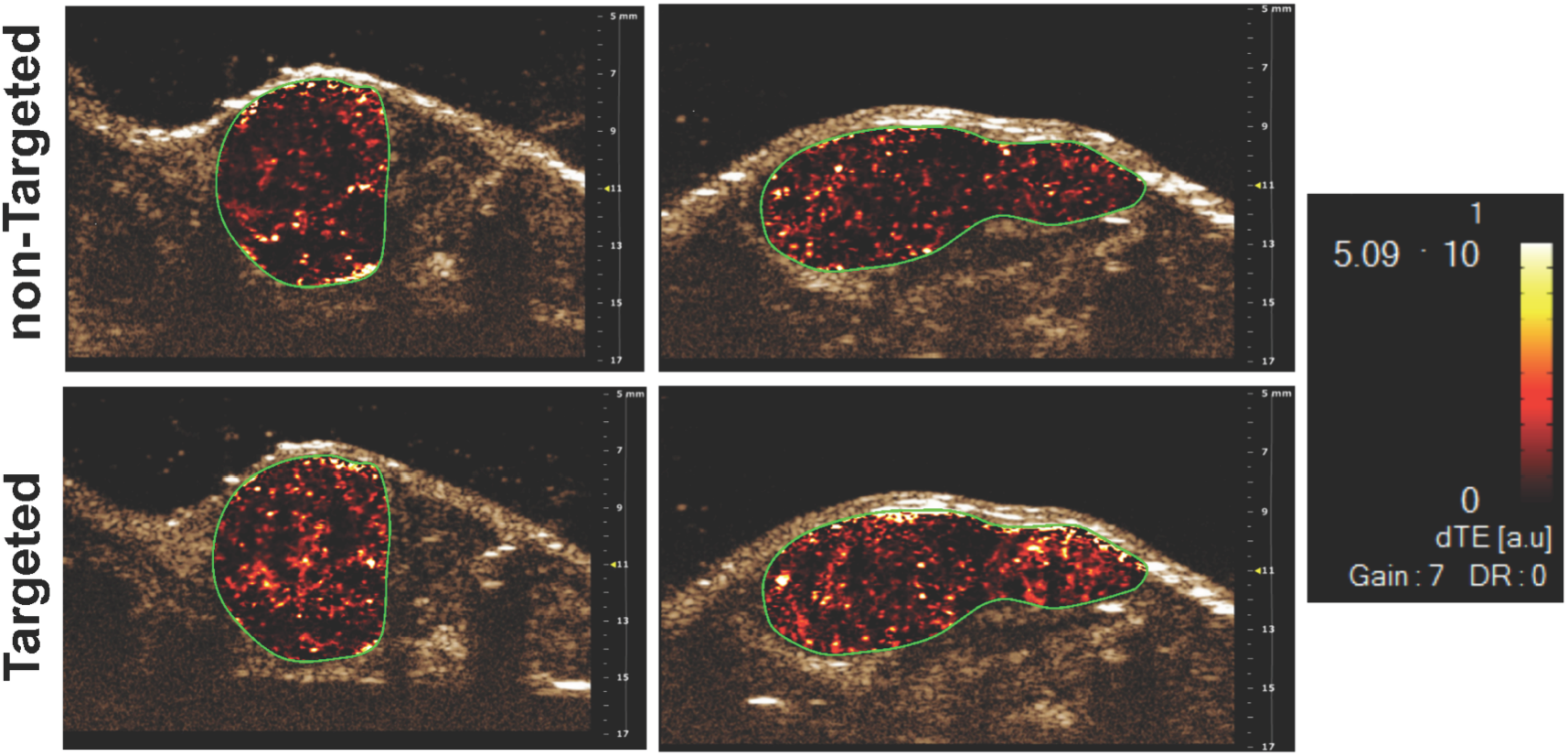
Representative US images showing targeted tumor localization of MB_Tz_ targeted with two different anti-PSMA antibodies. Direct targeting MB_Tz_ constructs are shown: MB_Tz-TCO_-J591 (left bottom) and MB_Tz-TCO_-ARP (right bottom). Images were first acquired 4 min after intravenous administration of non-targeted MB_Tz_ (top left and right), followed by the targeted constructs. Each pair of images (top/bottom) are from the same mouse and field of view. Images are transverse color-coded parametric images overlaid on a non-linear contrast mode US image with the whole LNCaP xenograft tumor (green outline) in the field of view. dTE = differential targeted enhancement. Complete imaging data can be found in S5 File.

**Fig 5:**
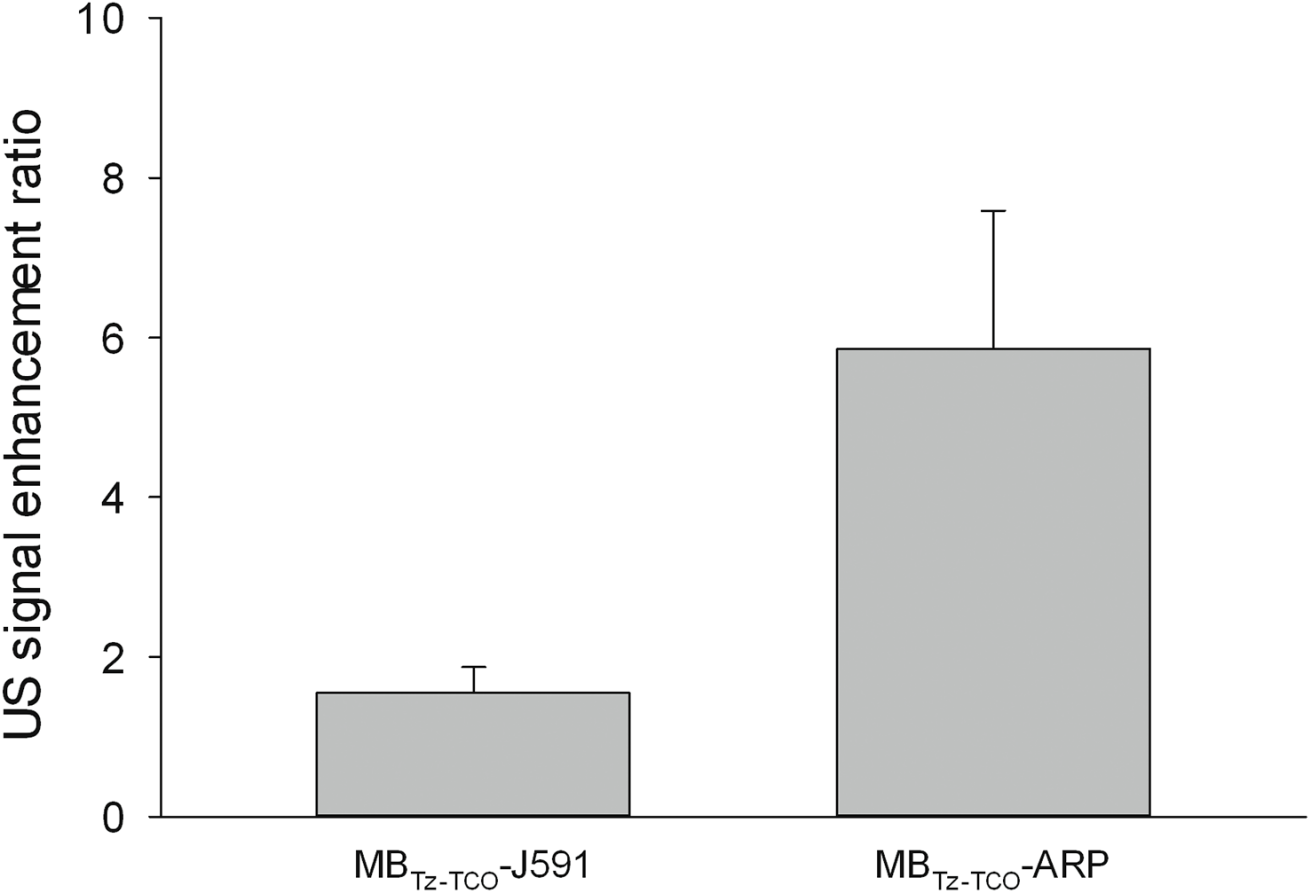
Comparison of the US signal enhancement obtained by direct targeting of PSMA^+^ LNCaP tumors using MB_Tz-TCO_-J591 and MB_Tz-TCO_-ARP. Data are the average ratio of US signals (n = 3, with SEM), for the targeting construct versus the control (MB_Tz_ alone). A statistically significant difference was not observed by one-way ANOVA (*P* = 0.07), due to variability of results from the mice injected with MB_Tz-TCO_-ARP.

For all studies, imaging was performed using the same plane of view as that for MBs containing no targeting agent (e.g. MB_Tz_ alone). Qualitatively, the parametric US images showed higher signal enhancement when using the MB_Tz-TCO_-J591 direct targeting construct, as compared to non-targeted MB_Tz_ (Fig 4). The quantified signal enhancement showed the mean of the ratio of signals for targeted to non-targeted MBs to be 1.6 ± 0.3 (Fig 5). This ratio of signal enhancement is comparable to that of previously reported PSMA-targeted nanobubbles [35,36]. The limited binding of MBs to the tumor is most likely due to the low human-PSMA expression in the vasculature in the human tumor, mouse xenograft model [58]. This correlates with a previous report suggesting that only mouse endothelial cells are present in the vasculature of human tumors grown in mice [59]. This would limit the effectiveness of J591 as a MB targeting molecule during preclinical studies since it only binds to human PSMA. Fortuitously, the fact that we employed Tz-TCO chemistry for preparing the targeted MBs makes it easy to vary the nature of the antibody. To this end, a second anti-PSMA antibody (ARP) that binds to both human and mouse PSMA was evaluated as a targeting vector. ARP was functionalized with TCO (TCO-ARP), loaded on MB_Tz_ and evaluated *in vivo* in the same model. When quantifying the signal enhancement compared to non-targeted MBs, the MB_Tz-TCO_-ARP construct showed 5.9 ± 1.7 fold enhancement (Fig 5). This was a larger signal enhancement than that observed for the ARP construct, although statistical analysis resulted in a *P* value (0.07) that did not reach significance. The apparent difference may indicate greater benefit for targeted US imaging in xenograft models through the use of antibodies that bind to both mouse and human PSMA. Irrespective of the differences in signal enhancement, both direct targeting constructs demonstrated enhanced accumulation of PSMA-targeted MBs in the tumor compared to the control non-targeted MB_Tz_. Furthermore, these results demonstrate the feasibility and ease of using bioorthogonal chemistry for screening different targeting vectors.

### *In vivo* US imaging of MBs in PSMA^+^ and PSMA^-^ PC-3 xenografts

**Fig 6:**
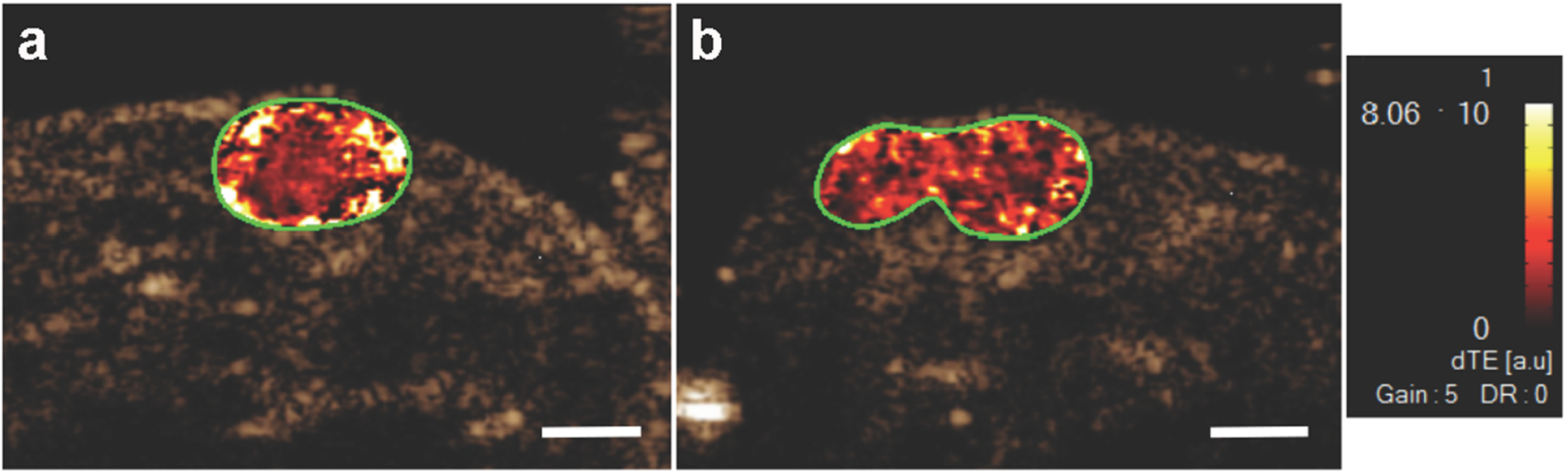
Representative US images showing targeted tumor localization of MB_Tz-TCO_-ARP in PC-3 tumor xenografts. The PSMA^+^ or PSMA^−^tumor were imaged 4 min after intravenous administration of MB_Tz-TCO_-ARP followed by imaging the other tumor. The images show qualitatively higher US signal in the PSMA^+^ tumor (a) compared to the signal found in the PSMA^−^tumor (b). Images are transverse color-coded parametric images overlaid on a non-linear contrast mode US image with whole PC-3 xenograft tumor (green outline) in the field of view. Scale bar = 2mm; dTE = differential targeted enhancement.

To evaluate the ability of the targeted MBs to differentiate between PSMA positive and negative tumors, an US imaging study was performed using MB_Tz-TCO_-ARP in SCID (male) mice that had both PSMA^+^ and PSMA^−^PC-3 tumors in each animal. This model was selected as it has been used to evaluate the ability of other molecular imaging probes [40] to differentiate between PSMA^+^ and PSMA^−^tumors. In this study, MB_Tz-TCO_-ARP was injected and allowed to circulate for 4 min. Using the disruption replenishment sequence, images were collected in a random order between the PSMA^+^ and PSMA^−^tumors. The parametric US images showed qualitatively higher signal enhancement in PSMA^+^ PC-3 tumors compared to PSMA^−^tumors (Fig 6).

**Fig 7:**
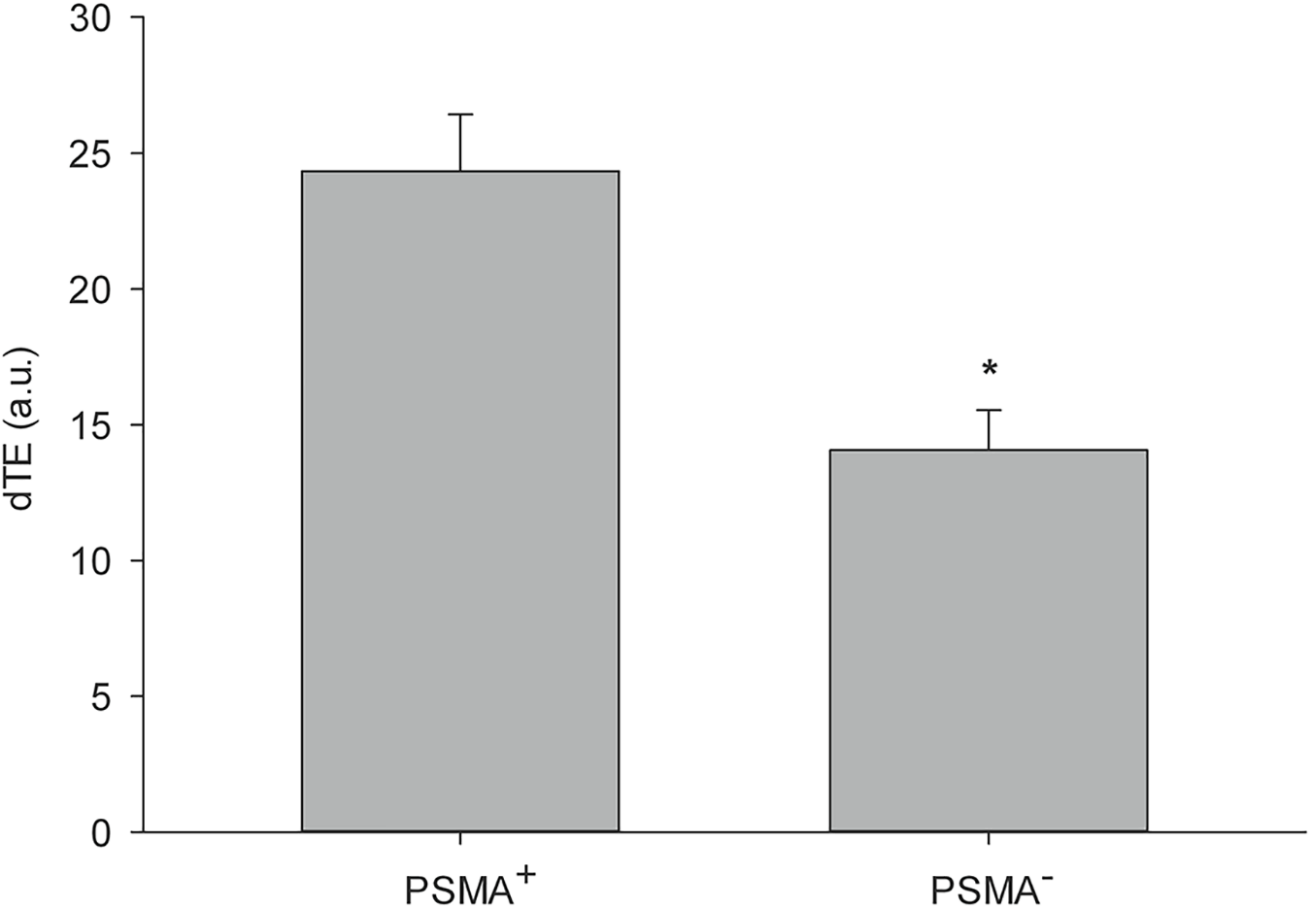
Differential targeted enhancement (dTE) signal obtained after accumulation of MB_Tz-TCO_-ARP in PC-3 xenograft tumors. Data are average signal enhancements obtained when imaging PSMA^+^ PC-3 tumors (n = 5) compared to PSMA^−^PC-3 tumors (n = 7). Signal obtained from accumulation of MB_Tz-TCO_-ARP in PSMA^+^ PC-3 tumors was significantly higher than that in PSMA^−^PC-3 tumors (*P* = 0.002). Statistical analysis was performed using one-way ANOVA.

The signal enhancement attributed to binding of MB_Tz-TCO_-ARP was then quantified and showed statistically significant higher signal (*P* = 0.002) in PSMA^+^ tumors (24.34 ± 2.08 a.u., n = 5) compared to that in PSMA^−^tumors (14.07 ± 1.47 a.u., n = 7) (Fig 7). Overall the accumulation of MB_Tz-TCO_-ARP in PSMA-expressing tumors was shown to be 1.97 fold higher (± 0.55) than that in PSMA-deficient tumors. These results indicate that the presence of PSMA promoted enhanced binding of the targeted MBs.

It is worth noting that these results show a larger signal enhancement for targeted MBs compared to the previous reports using PSMA-targeted nanobubbles [35,36]. For the latter, a statistically significant increase in signal intensity was observed when measuring US peak enhancement. However, those calculations included signal from both target bound and circulating nanobubbles, whereas in our approach a disruption replenishment sequence was used so that the quantified signal is only associated with bound MBs.

## Conclusions

The experiments demonstrated the effectiveness of TCO-Tz bioorthogonal chemistry for constructing US MBs that can bind to PSMA expressing tumors. Studies are ongoing to create a human compatible MB_Tz_ that when combined with a humanized anti-PSMA antibody (e.g. J591) will create PSMA-targeted MBs suitable for translation to clinical trials. This work will leverage the high yield of the Tz-TCO reaction and allow for production of targeted MBs with minimal modification to both the contrast agent and targeting moiety. While US imaging has a number of attractive features, the issues of user dependent variability and potential cost of the agent will need to be considered in the future when comparing the reported approach with other diagnostic methods. In the interim, the targeted MBs described here can be produced via straightforward chemistry and a commercially available MB kit. These PSMA-targeted MBs can be used for preclinical US imaging studies in animal models of PSMA-expressing cancers.

## Supporting information

S1 File MALDI-TOF MS of J591 and TCO-J591. (PDF)

S2 File Structure of biotin-tetrazine (biotin-Tz). (PDF)

S3 File Schematic diagram of the components and function of the parallel plate flow chamber. (PDF)

S4 File Western blot data for PSMA expression. (PDF)

S5 File US images showing PSMA-targeting of MB_Tz_ using two different PSMA antibodies. (PDF)
