# Supplementary material for: Development of prostate specific membrane antigen targeted ultrasound microbubbles using bioorthogonal chemistry
Source: PLoS One. 2017 May 4;12(5):e0176958. doi: 10.1371/journal.pone.0176958 (PMC5417523; doi:10.1371/journal.pone.0176958)
Supplement: S1 File — (PDF) [file pone.0176958.s001.pdf]

MALDI-TOF MS of J591 and TCO-J591

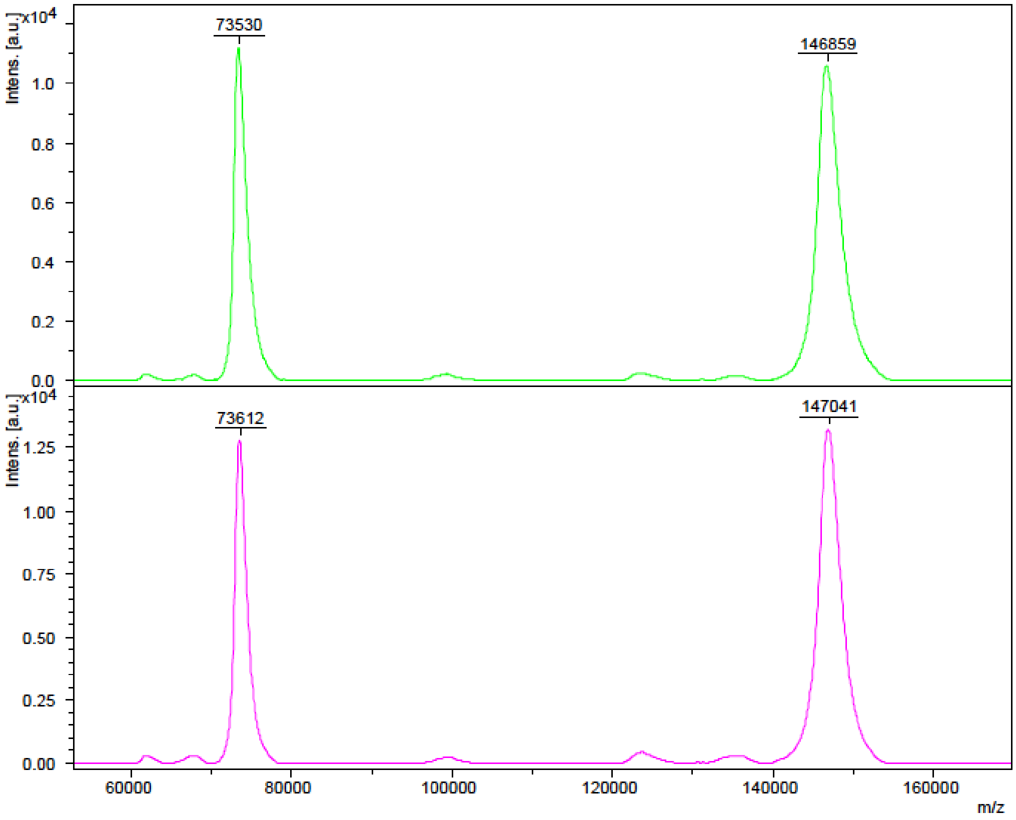

MALDI-TOF MS analysis of unconjugated J591 antibody (top) and TCO-conjugated J591 (bottom). The difference in molecular weight between the two samples indicated an average of 1.2 TCO molecules per antibody molecule for TCO-J591.
