# Supplementary figures and images for: Development of prostate specific membrane antigen targeted ultrasound microbubbles using bioorthogonal chemistry
Source: PLoS One. 2017 May 4;12(5):e0176958. doi: 10.1371/journal.pone.0176958 (PMC5417523; doi:10.1371/journal.pone.0176958)

## Structure of biotin-tetrazine (biotin-Tz)

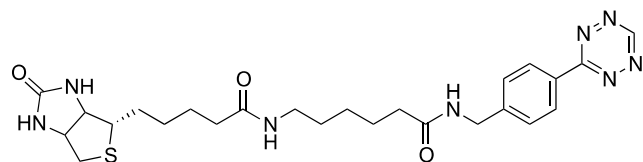

Supplement: S2 File — (PDF) [file pone.0176958.s002.pdf]
