# Supplementary material for: Development of prostate specific membrane antigen targeted ultrasound microbubbles using bioorthogonal chemistry
Source: PLoS One. 2017 May 4;12(5):e0176958. doi: 10.1371/journal.pone.0176958 (PMC5417523; doi:10.1371/journal.pone.0176958)
Supplement: S3 File — (PDF) [file pone.0176958.s003.pdf]

Schematic diagram of the components and function of the parallel plate flow chamber.

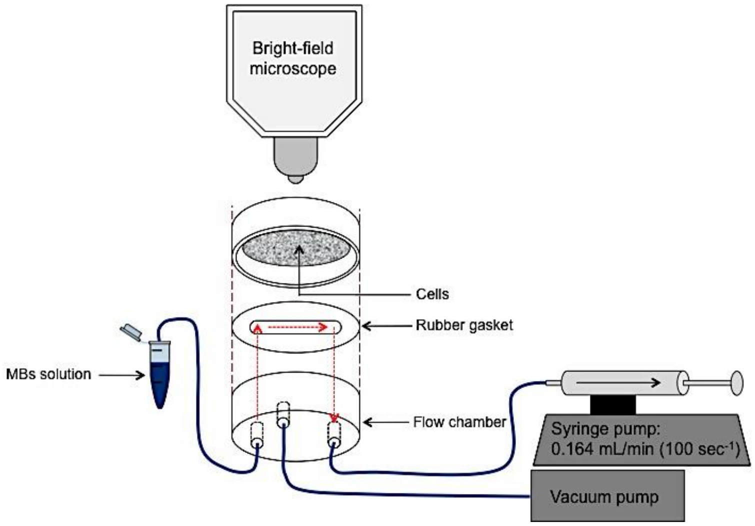

The parallel plate flow chamber system was used to test and visualize the binding of MBs to cultured cell lines under flow conditions [1]. The binding of MBs was assessed at a shear rate of 100 sec^{-1} which was produced by application of a syringe pump.

1. Zlitni A, Janzen N, Foster FS, Valliant JF. Catching Bubbles: Targeting Ultrasound Microbubbles Using Bioorthogonal Inverse-Electron-Demand Diels-Alder Reactions. Angew Chem Int Ed Engl 2014;53:6459–63.
