# Supplementary material for: Development of prostate specific membrane antigen targeted ultrasound microbubbles using bioorthogonal chemistry
Source: PLoS One. 2017 May 4;12(5):e0176958. doi: 10.1371/journal.pone.0176958 (PMC5417523; doi:10.1371/journal.pone.0176958)
Supplement: S4 File — (PDF) [file pone.0176958.s004.pdf]

Western blot data for PSMA expression.

1. Western blot analysis of PSMA protein expression in LNCaP and PC-3 cell lysates.

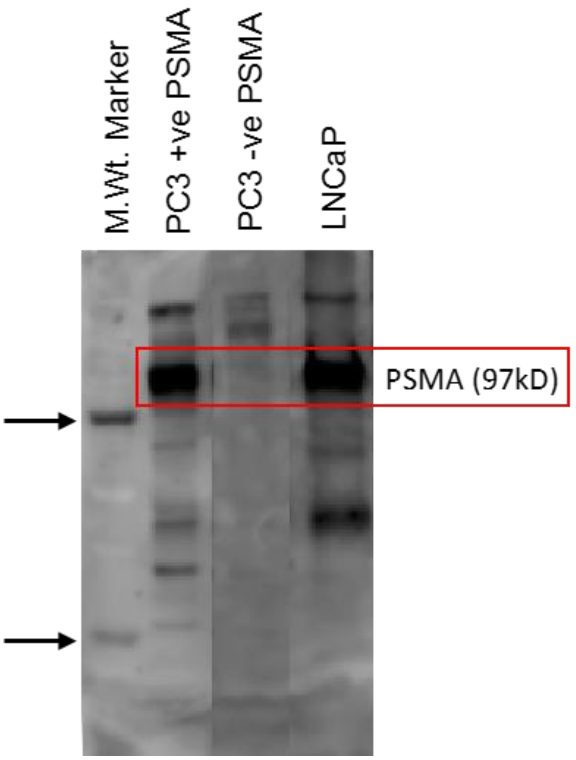

Cell lysates were prepared from PSMA transfected (+ve) PC-3 cells, PSMA (-ve) PC-3 cells, and LNCaP cells grown in culture. Lysate proteins were separated by SDS-PAGE. Western blots were performed using polyclonal goat anti-human PSMA antibody. Arrows indicate MW markers (75 and 25 kD). PSMA band is indicated at 97kD.

2. Western Blot analysis of PSMA expression in LNCaP tumor lysates.

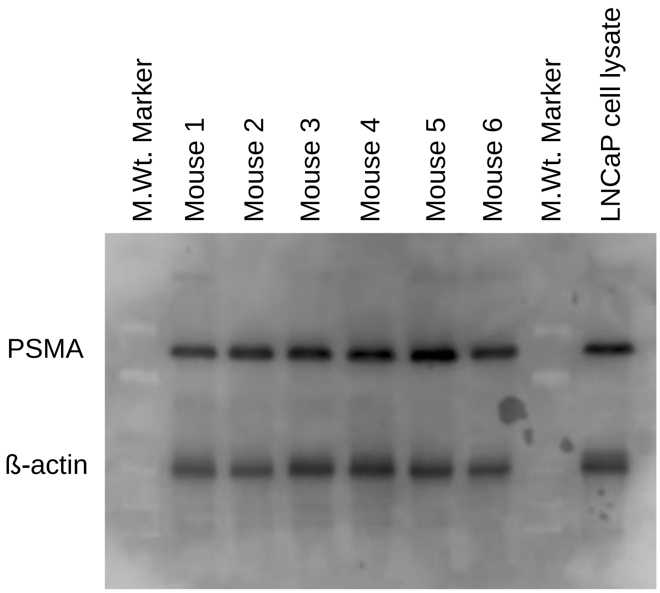

LNCaP tumor lysates from the *in vivo* imaging studies were prepared and proteins separated by SDS-PAGE, followed by western blot analysis and immunostaining for PSMA and β-actin. Top band indicates PSMA, and bottom band β-actin. Lysate from LNCaP cells grown *in vitro* are shown on the right for comparison.

3. Quantitative comparison of PSMA expression in LNCaP tumors

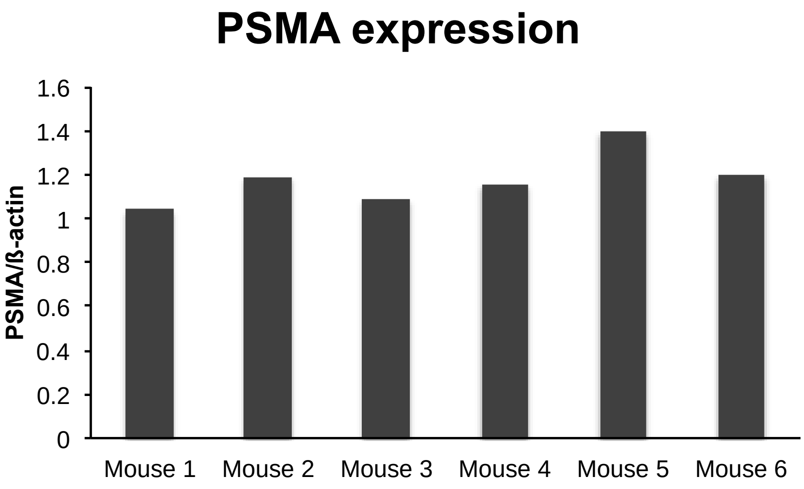

PSMA protein expression measured by band image density from western blots of LNCaP tumor lysates from 6 mice, and normalized to ß-actin band image density. No significant difference in

PSMA expression was found among six tumors (image analysis was done using ImageQuant TL software).
