# Supplementary material for: Development of prostate specific membrane antigen targeted ultrasound microbubbles using bioorthogonal chemistry
Source: PLoS One. 2017 May 4;12(5):e0176958. doi: 10.1371/journal.pone.0176958 (PMC5417523; doi:10.1371/journal.pone.0176958)
Supplement: S5 File — (PDF) [file pone.0176958.s005.pdf]

US images showing PSMA-targeting of MB_{Tz} using two different PSMA antibodies:

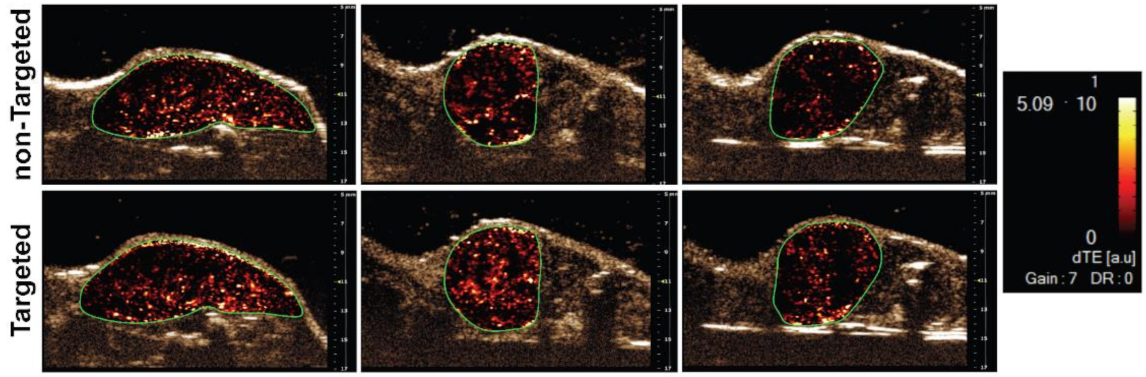

US images showing localization of $MB_{Tz\text{-}TCO}$-J591 to LNCaP tumors.
LNCaP xenograft tumors (green outline) were produced in mice as described in methods. Transverse color-coded parametric images overlaid on a nonlinear contrast mode ultrasound images were acquired 4 min after intravenous administration of either control non-targeted MB_{Tz} (top) or direct PSMA-targeted $MB_{Tz\text{-}TCO}$-J591 (bottom). Signals are indicated by dTE = differential targeted enhancement (color scale, right).

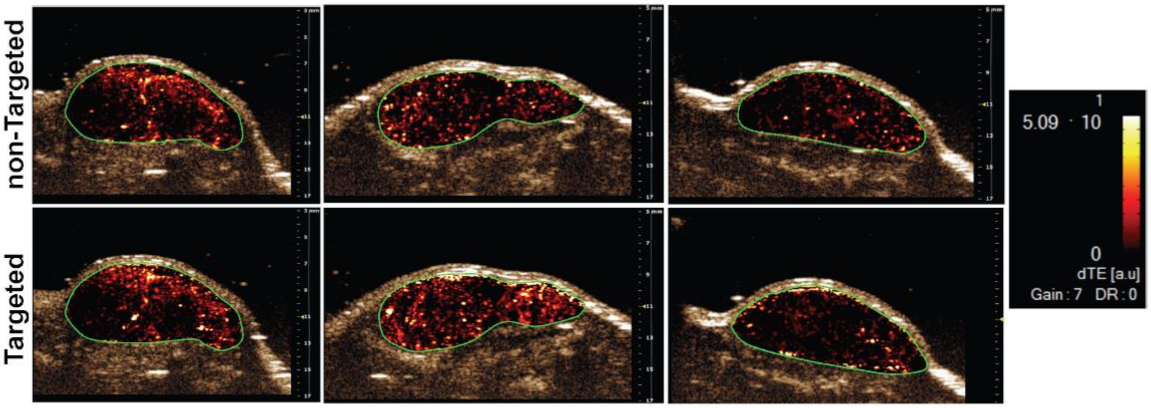

US images showing localization of $MB_{Tz\text{-}TCO}$-ARP to LNCaP tumors.
LNCaP xenograft tumors (green outline) were produced in mice as described in methods. Transverse color-coded parametric images overlaid on a nonlinear contrast mode ultrasound images were acquired 4 min after intravenous administration of either control non-targeted MB_{Tz} (top) or direct PSMA-targeted $MB_{Tz\text{-}TCO}$-ARP (bottom). Signals are indicated by dTE = differential targeted enhancement (color scale, right).
